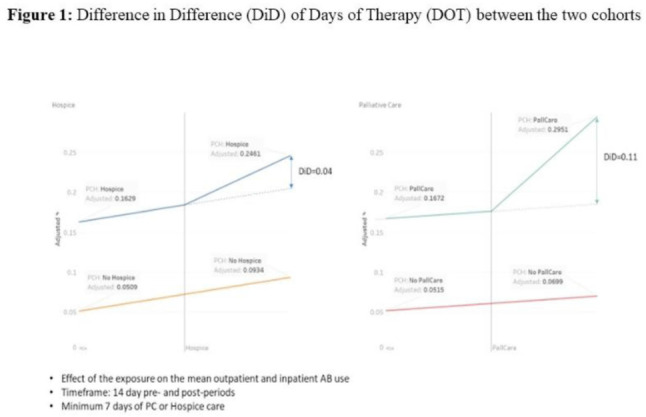# Antibiotic use in end-of-life care patients: A nationwide Veterans’ Health Administration cohort study

**DOI:** 10.1017/ash.2022.89

**Published:** 2022-05-16

**Authors:** Alexandre Marra, Gosia Clore, Erin Balkenende, Cassie Goedken, Daniel Livorsi, Michihiko Goto, Vaughan-Sarrazin, Ann Broderick, Eli Perencevich

## Abstract

**Background:** Antibiotic use during end-of-life (EOL) care is an increasingly important target for antimicrobial stewardship given the high prevalence of antibiotic use in this setting with limited evidence on safety and effectiveness to guide antibiotic decision making. We estimated antibiotic use during the last 6 months of life for patients under hospice or palliative care, and we identified potential targets (ie time points) during the EOL period when antimicrobial stewardship interventions could be targeted for maximal benefit. **Methods:** We conducted a retrospective cohort study of nationwide Veterans’ Affairs (VA) patients, 18 years and older who died between January 1, 2014, and December 31, 2019, and who had been hospitalized within 6 months prior to death. Data from the VA’s integrated electronic medical record (EMR) were collected including demographics, comorbid conditions, and duration of inpatient antibiotics administered, along with outpatient antibiotics dispensed. A propensity-score matched-cohort analysis was conducted to compare antibiotic use between patients placed into palliative care or hospice matched to patients not receiving palliative care or hospice care. Repeated measures ANOVA and repeated measures linear regression methods were used to analyze difference in difference (D-I-D) of days of therapy (DOT) between the 2 cohorts. **Results:** There were 251,822 patients in the cohort, including 23,746 in hospice care, 89,768 in palliative care, and 138,308 without palliative or hospice care. The median days from last discharge to death was 9 days. The most common comorbidities were chronic obstructive pulmonary diseases (50%), malignancy (46%), and diabetes mellitus (43%). Overall, 18,296 (77%) of 23,746 hospice patients, and 71,812 (80%) of 89,768 palliative care patients received at least 1 antibiotic, whereas 95,167 (69%) of 138,308 who were not placed in hospice or did not receive palliative care received antibiotics. In the primary matched cohort analysis that compared patients placed into hospice or palliative care to propensity-score matched controls, entry into palliative care was associated with a 11% absolute increase in antibiotic prescribing, and entry into hospice was associated with a 4% absolute increase during the 7–14 days after entry versus the 7–14 days before entry (Fig. 1). The stratified cohorts had very similar balanced covariates as the overall cohort. **Conclusions:** In our large cohort study, we observed that patients receiving EOL care had high levels of antibiotic exposure across VA population, particularly on entry to hospice or during admissions when they received palliative care consultation. Future studies are needed to identify the optimal EOL strategies for collaboration between antimicrobial stewardship and palliative care.

**Funding:** None

**Disclosures:** None

**Figure:**